# Playground usage and physical activity levels of children based on playground spatial features

**DOI:** 10.1007/s10389-017-0828-x

**Published:** 2017-09-04

**Authors:** Anne K. Reimers, Guido Knapp

**Affiliations:** 1 Institute of Human Movement Science and Health, Faculty of Behavioral and Social Sciences, Chemnitz University of Technology, Straße der Nationen 62, 09111 Chemnitz, Germany; 2 Faculty of Statistics, Technische Universität Dortmund, 44221 Dortmund, Germany

**Keywords:** Physical activity, Playgrounds, Play facilities, Children, Audit instrument, Direct observation

## Abstract

**Aim:**

Being outdoors is one of the strongest correlates of physical activity in children. Playgrounds are spaces especially designed to enable and foster physical activity in children. This study aimed to analyze the relationship between the spatial features of public playgrounds and the usage and physical activity levels of children playing in them.

**Subjects and methods:**

A quantitative, observational study was conducted of ten playgrounds in one district of a middle-sized town in Germany. Playground spatial features were captured using an audit instrument and the playground manual of the town. Playground usage and physical activity levels of children were assessed using a modified version of the System for Observing Play and Leisure Activity in Youth. Negative binomial models were used to analyze the count data.

**Results:**

The number of children using the playgrounds and the number of children actively playing in them were higher in those with more varied facilities and without naturalness. Girls played more actively in playgrounds without multi-purpose areas. Cleanliness, esthetics, play facility quality, division of functional areas and playground size were not related to any outcome variable.

**Conclusion:**

Playground spatial features are related to playground usage and activity levels of the children in the playgrounds. Playgrounds should offer a wide variety of play facilities and provide spaces for diverse play activities to respond to the needs of large numbers of different children and to provide activity-friendly areas enabling their healthy development.

## Introduction

Physical activity in children and adolescents is the basis of growing up healthily. Furthermore, lifestyle habits are developed in the course of childhood and adolescence and may persist into adulthood (Rauner et al. 2015). Many studies have shown that physically active children and adolescents are more physically active throughout their lifespan than their inactive counterparts (Telama 2009). Nevertheless, many children worldwide are not meeting physical activity guidelines and are not active enough to grow up healthily (Hallal et al. 2012). In Germany, only 13.1% of girls and 17.4% of boys aged 4–17 years old meet the physical activity guidelines (Jekauc et al. 2012).

According to social cognitive theory (Bandura 1986), both social and physical environments influence behavior and vice versa. A wide range of studies has shown that the neighborhood environment is an essential physical context, having a large impact on physical activity patterns of children and adolescents (de Vet et al. 2011; Ding et al. 2011). Furthermore, being outside is one of the strongest determinants of physical activity in youth: an observational study with children from low- to middle-income families in San Diego, CA, USA, for example, showed that the strongest correlate of children’s physical activity at home was time spent outdoors (Sallis et al. 1993).

Being outdoors seems to be a necessary precondition for many physical activities since some games and activities, like playing tag or tennis, cannot take place indoors in a children’s room. The wide space that is provided when being outdoors in parks, forests, inner courtyards, traffic-calmed streets, play areas or playgrounds facilitates a larger range of activities and more intensive physical activity. Being outside with a lower risk of injury and/or damaging objects may also lead to greater physical activity opportunities for children and adolescents. However, high-speed, high-volume traffic on the roads, limited pedestrian safety structures, walking or biking facilities and restricted access to recreational facilities all hinder outdoor physical activity in children and adolescents (Ding et al. 2011; Scott et al. 2007). Physical activity spaces in the neighborhood must be available to facilitate outdoor physical activity in children and adolescents.

Playgrounds are places specifically developed to offer opportunities for children to play and be physically active, thus facilitating healthy development (Broekhuizen et al. 2014). With various play equipment and play areas, they provide affordances that do not necessarily cause children’s activities, but which offer action possibilities. Broekhuizen et al. (2014) conducted a systematic review of the value of (pre-)school playgrounds on children’s physical activity levels and found positive associations between provision of (portable and fixed) play equipment, playground size and decreased playground density (children per square meter) on physical activity in school children. In another review, the contribution of playground designs to increasing physical activity levels was also investigated in a school setting and showed that playground markings plus physical structures increase physical activity during recess (Escalante et al. 2014). Nevertheless, few studies have been conducted to investigate which features and characteristics of neighborhood, out-of-school playgrounds foster usage and physical activity levels in children and how playgrounds should be designed to promote physical activity out of school.

Moreover, different physical activity patterns in boys and girls have never been considered in relation to playground characteristics. Girls and boys are not physically active at the same level; they have different physical activity habits and show different behaviors when using available activity spaces: Fewer girls meet physical activity guidelines compared with boys (Hallal et al. 2012); girls tend to rarely choose ball games (Wilson et al. 2005), and they occupy less space than boys (Karsten 2003). Furthermore, girls have less independent mobility than boys (Brown et al. 2008; Page et al. 2009) and thus may have less access to play and activity areas in the neighborhood (Reimers et al. 2014). For example, an observational study of eight playgrounds in Amsterdam showed that in all playgrounds the greater proportion of users was male, with girls comprising only one-third of all children observed at the playgrounds (Karsten 2003). Additionally, this study revealed that girls favored playgrounds with lots of play facilities and in a good condition. In conclusion, girls may have different play facility and equipment demands and may use playgrounds in a different way than boys (Colabianchi et al. 2011; Karsten 2003).

Thus, the aim of the current study was to investigate: (1) how hardware characteristics and qualitative aspects of playgrounds contribute to usage of playgrounds by children, (2) how they influence their physical activity levels and (3) whether there are any gender differences concerning these research questions.

## Methods

### Study design

This quantitative, observational study was conducted in a district of Constance (Petershausen-West), which is a middle-sized town (83,000 inhabitants) in the south of Germany, directly on the border to Switzerland. In 2013, approximately 22,000 inhabitants lived in this district, of whom around 3000 were children and adolescents between 0 and 17 years old (City of Constance 2013), which indicates a high proportion of minor citizens in relation to other districts in Constance. The district has a total surface of 181.89 ha and is densely populated (124.14 inhabitants per ha). Eighty-three percent of the inhabitants have German nationality, and 3.6% are unemployed (by comparison, the overall unemployment rate of Constance is 2.8%). Ten playgrounds in Petershausen-West were selected based on their location (agglomeration of many children and adolescents living close to the playgrounds) and their target group (playgrounds made for children and adolescents).

### Observation schedule and protocol

To capture aspects of the playgrounds and their usage as well as the physical activity levels of users, an audit tool and a direct observation tool were selected. In March 2015, trained observers conducted an audit to assess the hardware characteristics and qualitative aspects of the selected playgrounds by actively inspecting them. To check the test-retest reliability of the audit tool, each playground was examined by two independent observers.

The SOPLAY (System for Observing Play and Leisure Activity in Youth) manual (McKenzie 2006) was used to guide data collection procedures of physical activity and playground usage. Three observers were initially trained and pilot tested the observation instrument in March 2015. Observer training comprised classroom lectures and pilot testing in the field. After pilot testing, the trained observers visited the playgrounds from April to September 2015 for investigation periods lasting 1 h between 10 a.m. and 8 p.m. to conduct direct observations based on a momentary time sampling procedure. On school days the observations took place between 12 noon and 8 p.m. and during the summer holiday period (July 30 to September 13, 2015) between 10 a.m. and 8 p.m.

Playground usage and physical activity levels of the users of the playgrounds were examined by means of scans. During these scans, the observer let his or her eyes wander from one side to the other of the playground. To facilitate counting of the number of users or the users’ physical activity levels, a mechanical counter was used (McKenzie 2006). Each scan encompassed six of these eye sweeps over the playground. In the first sweep, the number of female users within an age group was counted after the observers had assigned the playground users to an age group by visual assessment. The following sweeps were conducted to count the number of female children in relation to their physical activity level (sedentary, walking or intensive) and the number of female adolescents in relation to their physical activity level. Afterwards this procedure was repeated for male users. In the current study, children were assigned to the children’s group when the estimated age was 12 or below.

To control for time and observers, all playgrounds were equally examined by all observers, and the investigation hours were evenly distributed over the day (between 10 a.m. and 8 p.m. during the holidays and between 12 noon and 8 p.m. during schooltimes). During each hour of observation, the observation procedure was repeated up to four times.

During these periods, the observers had a fixed position in the playground (e.g., a centrally located bench) from which observation of the whole playground was possible. To avoid reactivity, the observers behaved as unobtrusively as possible. If so requested by parents or supervisors, the observers offered an informative letter containing details of the aims and procedure of the study, the responsible researchers and institutions, and data security measures. Observers also provided verbal information about their data collection in the playground and about the study when requested.

### Measures

#### Playground spatial features

To measure the provision and quality of playground features, an audit tool was developed based on that of Jones et al. (2010) and the Environmental Assessment of Public Recreation Spaces (EAPRS) (Saelens et al. 2006). It contained categories on play facility provision, play facility quality, esthetics and cleanliness of play facilities, with each comprising multiple items covering areas such as things to hang from, things to slide down, things to climb up, things to climb through, things to stand/walk on, swings, carousels, see-saws, playing fields and sandpits. In the play facility provision category, for example, the items were: “Are there any things to hang from?”, “Are there any things to slide down from?”, etc. The response options for the playground feature scales were based on dichotomous answers (e.g., “Are there any swings?”—yes/no) or rating scales with three (e.g., “What condition are the swings in?”—good, satisfactory, bad) or five points (e.g., “Is the playground free from vandalism?”—responses ranging from strongly agree to strongly disagree). To calculate composite scores, the values of individual items were summarized and a mean score was computed.

Additionally, further variables were selected from the playground manual of the City of Constance (Schmitz et al. 2008), which describes all playgrounds in Constance and rates their conditions. Provision of multi-purpose areas, naturalness and division of functional spaces were coded dichotomously (yes/no). Playground size was given in square meters and the size of the catchment area as a categorical variable (neighborhood, quarter or district). Provision of multi-purpose spaces was coded as yes if there were multi-purpose areas like lawns or pavements that could be used for playing on. Naturalness was coded as yes if the playground had natural design elements and/or was planted. A division of functional areas was assumed if playground areas for different target groups or different functions were spatially divided.

#### Playground usage and physical activity levels

To capture the usage of playgrounds and the physical activity levels of playground users within a defined time period by direct observation, a modified SOPLAY version (McKenzie et al. 2000) was used. With the direct observation tool, the number of users per age group and the number of children featuring a physical activity level (sedentary, walking or intensive) were examined separately for males and females. Gender and age groups were rated by the observers through visual assessment. Children who were walking or conducting intensive activities were assigned to the moderate-to-vigorous physical activity (MVPA) group. This study was based on children only.

#### Confounding factors

Daily weather (temperature during the hour of observation, rainfall) was captured as a confounding factor by using weather protocols provided by two online German weather services (www.wetterspiegel.de and www.wetterkontor.de).

#### Statistical analysis

All analyses were conducted with SAS Software 9.4 (SAS Corp., Cary, NC, USA) and were based on the numbers of children (not adolescents and adults) using the playgrounds. Descriptive statistics were conducted for all independent variables and for all outcome variables. The outcome variables relating to the number of children using the playground and the number of children in the MVPA group were quantified as count variables. Play facility provision, play facility quality, esthetics, cleanliness of play facilities, naturalness, division of functional areas, provision of multi-purpose areas and playground size were considered as independent variables.

A negative binomial model was a better fit for the data than a Poisson model with overdispersion or a zero-inflated Poisson model assuming independence of all observations. The independence assumption clearly holds for observations from different playgrounds and may be justified for observations on different days in the same playground. However, some observations (scans) were done within an hour in the same playground. Consequently, all observations carried out on the same day in the same playground are correlated and form a cluster. In total, we had 194 clusters consisting of 1–9 observations. Therefore, we opted for a generalized estimating equation (GEE) approach using negative binomial distribution. For the working correlation structure, we used the compound symmetry (exchangeable) correlation structure because of the relatively short total observation periods. Statistical significance was considered at *p* < 0.05.

## Results

A total of 578 scans were conducted, and all playgrounds were visited for observation 18–21 times. Of the ten study playgrounds, one had a neighborhood-level catchment area, six a quarter-level catchment area and three a district-level catchment area. The size of the playgrounds ranged from 280 m² to 3800 m². The descriptive statistics on playground features are provided in Table 1. The number of playground users across all scans ranged from 0 to 43 (Table 2). In total, 2538 children (1284 girls and 1254 boys) were observed in the playgrounds. The mean number of children in any given playground was 4.39 (SD = 5.11) and ranged between 0.94 and 10.08. The mean number of active children was 2.24 (SD = 2.82) and ranged between 0.12 and 4.91. On average, about the same number of girls and boys played in the playgrounds (2.22 ± 2.82 and 2.17 ± 2.70, respectively). The mean number of children being at least moderately physically active was 1.03 (SD = 1.58) girls and 1.21 (SD = 1.68) boys. On average, 2.10 (SD = 2.97) children in the playgrounds were sedentary.

**Table 1:** Descriptive statistics of playground features (*N* = 10)

Variable (possible score range)	Mean ± SD or % yes
Esthetics (0–14)	6.80 ± 1.09
Cleanliness (0–2)	1.62 ± 0.28
Provision of play facilities (0–10)	5.50 ± 1.96
Play facility quality (0–10)	8.80 ± 3.74
Naturalness (yes/no)	50
Division of functional areas (yes/no)	40
Provision of multi-purpose areas (yes/no)	60
Playground size (in m²)	1753 ± 1163

*SD* standard deviation

**Table 2:** Number of children using playgrounds and number of children per physical activity level (MVPA, sedentary)

	Number of observations with 0 children in playgrounds (% of all observations); (total number of observations: *N* = 578)	Max. number of children	Number of children in playgrounds per observation (Mean ± SD)
Playground users	159 (28%)	36	4.39 ± 5.11
Girls	228 (39%)	19	2.22 ± 2.82
Boys	197 (34%)	18	2.17 ± 2.70
Physical activity levels			
Sedentary	221 (38%)	24	2.10 ± 2.97
Girls	295 (51%)	17	1.15 ± 1.89
Boys	322 (56%)	10	0.95 ± 1.51
MVPA	229 (40%)	25	2.24 ± 2.82
Girls	320 (55%)	14	1.03 ± 1.58
Boys	283 (49%)	11	1.21 ± 1.68

*MVPA* moderate-to-vigorous physical activity, *SD* standard deviation

Statistical results on the relationship between playground features and usage and physical activity levels in children and girls and boys, respectively, are presented in Tables 3, 4 and 5. Overall, the numbers of children present in playgrounds and the numbers of children being at least moderately active were higher in playgrounds providing more varied play facilities (*B* = 0.54, 95% CI: 0.23–0.84; *p* < 0.001; *B* = 0.53, 95% CI: 0.19–0.86; *p* = 0.001) and in playgrounds without naturalness (*B* = −0.91, 95% CI: −1.55–−0.28; *p* = 0.005; *B* = −0.74, 95% CI: −1.46–−0.02; *p* = 0.045). None of the other variables predicted playground usage or physical activity levels in children in a multivariate model when disregarding gender. In girls (Table 4), play facility provision and naturalness predicted playground usage (*B* = 0.46, 95% CI: 0.12–0.80; *p* = 0.008; *B* = −0.74, 95% CI: −1.46–−0.03; *p* = 0.043), and play facility provision also predicted the number of girls being at least moderately physically active (*B* = 0.43, 95% CI: 0.05–0.81; *p* = 0.028). Furthermore, in playgrounds with no provision of multi-purpose areas, girls were more likely to be moderately physically active than in playgrounds with multi-purpose areas (*B* = −2.61, 95% CI: −4.89–0.33; *p* = 0.025). For boys, the number of users was higher in playgrounds providing more varied play facilities and without naturalness than in natural playgrounds with fewer types of play facilities. The results did not crucially differ in models without adjustment for rainfall and temperature.

**Table 3:** Generalized estimating equation (GEE) for predicting playground usage and number of children being physically active in playgrounds (MVPA)

	Playground usage						MVPA					
			95% CI for *B*						95% CI for *B*			
	*B*	*SE*	Lower	Upper	*Z*	*p*	*B*	*SE*	Lower	Upper	*Z*	*p*
Intercept	−0.77	1.37	−3.46	1.91	−0.56	0.574	−3.05	1.47	−5.93	−0.17	−2.07	0.038
Esthetics	−0.23	0.28	−0.77	0.31	−0.82	0.410	−0.10	0.32	−0.72	0.53	−0.30	0.761
Cleanliness	−0.55	0.73	−1.98	0.88	−0.75	0.452	0.25	0.94	−1.58	2.09	0.27	0.786
Play facility provision	0.54	0.15	0.23	0.84	3.49	<0.001	0.53	0.17	0.19	0.86	3.08	0.002
Play facility quality	0.11	0.07	−0.01	0.24	1.74	0.082	0.10	0.07	−0.05	0.24	1.35	0.178
Naturalness	−0.91	0.32	−1.55	−0.28	−2.83	0.005	−0.74	0.37	−1.46	−0.02	−2.01	0.045
Division of functional areas	1.10	0.64	−0.16	2.36	1.71	0.087	0.74	0.77	−0.77	2.25	0.96	0.338
Provision of multi-purpose areas	−1.36	0.88	−3.08	0.36	−1.55	0.121	−1.65	0.98	−3.57	0.27	−1.68	0.092
Playground size	0.00	0.00	−0.02	0.00	0.73	0.464	0.00	0.00	−0.00	0.00	0.66	0.512

All models were adjusted for weather (temperature and rainfall); *N* = 578

Playground usage: QIC = −2384; MVPA: QIC = −196

*B* unstandardized regression weight, *SE* standard error, *Z* z-score; *p* probability value, *MVPA* moderate-to-vigorous physical activity, *QIC* quasi-likelihood under independence model criterion

**Table 4:** Generalized estimating equation (GEE) for predicting playground usage in girls and number of girls being physically active in playgrounds (MVPA)

	Playground usage						MVPA					
			95% CI for *B*						95% CI for *B*			
	*B*	*SE*	Lower	Upper	*Z*	*p*	*B*	*SE*	Lower	Upper	*Z*	*p*
Intercept	−2.32	1.51	−5.28	0.65	−1.53	0.125	−6.09	1.90	−9.81	−2.36	−3.20	0.001
Esthetics	−0.13	0.32	−0.76	0.50	−0.40	0.687	0.07	0.386	−0.68	0.83	0.19	0.852
Cleanliness	−0.46	1.00	−2.42	1.50	−0.46	0.646	0.64	1.10	−1.52	2.80	0.58	0.561
Play facility provision	0.46	0.17	0.12	0.80	2.66	0.008	0.43	0.20	0.05	0.81	2.20	0.028
Play facility quality	0.13	0.07	−0.02	0.27	1.74	0.083	0.16	0.09	−0.01	0.33	1.84	0.066
Naturalness	−0.74	0.37	−1.46	−0.03	−2.03	0.043	−0.01	0.48	−0.95	0.93	−0.02	0.987
Division of functional areas	0.87	0.81	−0.72	2.47	1.07	0.285	−0.18	0.93	−2.00	1.65	−0.19	0.849
Provision of multi-purpose areas	−1.59	0.97	−3.50	0.32	−1.64	0.102	−2.61	1.16	−4.89	−0.33	−2.24	0.025
Playground size	0.00	0.00	−0.00	0.00	0.81	0.417	0.00	0.00	−0.00	0.00	1.35	0.178

All models were adjusted for weather (temperature and rainfall); *N* = 578

Playground usage: QIC = −176; MVPA: QIC = 531

*B* unstandardized regression weight, *SE* standard error, *Z* z-score, *p* probability value, *MVPA* moderate-to-vigorous physical activity, *QIC* quasi-likelihood under independence model criterion

**Table 5:** Generalized estimating equation (GEE) for predicting playground usage in boys and number of boys being physically active in playgrounds (MVPA)

	Playground usage						MVPA					
			95% CI for *B*						95% CI for *B*			
	*B*	*SE*	Lower	Upper	*Z*	*p*	*B*	*SE*	Lower	Upper	*Z*	*p*
Intercept	−1.11	1.52	−4.08	1.86	−0.73	0.465	−2.29	1.62	−5.45	0.88	−1.41	0.158
Esthetics	−0.44	0.33	−1.09	0.21	−1.33	0.184	−0.22	0.34	−0.88	0.45	−0.63	0.527
Cleanliness	−0.42	0.82	−2.03	1.19	−0.51	0.611	−0.17	1.08	−2.30	1.95	−0.16	0.874
Play facility provision	0.70	0.20	0.32	1.08	3.58	<0.001	0.65	0.19	0.28	1.02	3.45	<0.001
Play facility quality	0.14	0.08	−0.02	0.29	1.73	0.084	0.07	0.08	−0.08	0.23	0.92	0.356
Naturalness	−1.07	0.38	−1.81	−0.33	−2.82	0.005	−1.30	0.41	−2.10	−0.50	−3.20	0.001
Division of functional areas	1.12	0.72	−0.30	2.54	1.54	0.123	1.45	0.86	−0.23	3.13	1.69	0.091
Provision of multi-purpose areas	−1.67	1.07	−3.76	0.43	−1.56	0.118	−1.18	1.04	−3.22	0.86	−1.13	0.258
Playground size	0.00	0.00	−0.00	0.00	0.79	0.428	0.00	0.00	−0.00	0.00	0.00	0.997

All models were adjusted for weather (temperature and rainfall); *N* = 578

Playground usage: QIC = −86; MVPA: QIC = 563

*B* unstandardized regression weight, *SE* standard error, *Z* z-score, *p* probability value, *MVPA* moderate-to-vigorous physical activity, *QIC* quasi-likelihood under independence model criterion

## Discussion

The current research project aimed to investigate the relationship between playground features and usage and the physical activity levels of children in playgrounds, also taking gender differences into consideration. We were able to show that the provision of a variety of play facilities fostered playground usage in children and that playgrounds without naturalness were visited more frequently by children than natural playgrounds. This is one of the first studies examining the relationship between playground features and children’s usage and activity levels in public playgrounds using direct observation techniques and audit instruments. Previous studies have all focused particularly on (primary) school playgrounds (Colabianchi et al. 2011; Dyment and O’Connell 2013; Kelly et al. 2012; Möhrle et al. 2015).

Our study showed that girls and boys played in playgrounds in equal numbers and that there was not much difference in the number of active girls and boys. Girls seemed to have equal access to playgrounds and were as likely to use them as boys. This is surprising, as in Germany overall physical activity levels are lower in girls than in boys (Jekauc et al. 2012; Kettner et al. 2013). Furthermore, this finding is in contrast to those of Karsten (2003) who observed children’s behavior in public playgrounds in Amsterdam and found many more boys visiting the playgrounds. It has been stated that girls are less likely to go to playgrounds because they have more restricted independent mobility than boys (Brown et al. 2008). In 1991, Shilling also posited that girls may feel intimidated by boys as they “draw on patriarchal ‘rules’ and ‘resources’ in using space as a way of asserting dominance over girls” (Shilling 1991). Nevertheless, the gender-specific role behavior of girls in playgrounds has changed during recent decades, and today girls behave more like boys, as shown in our study.

It transpired that playground features did not prevent girls from using the playgrounds in comparison to boys, but we did find that different playground features predicted physical activity levels in boys and girls. For example, we found fewer active girls in playgrounds providing multi-purpose areas utilizable for ball games. This finding is consistent with the results of Bocarro et al. (2012) who found notably more boys playing in multi-purpose areas or in football/soccer areas than girls in school playgrounds. Perhaps in our study girls were less active in playgrounds providing multi-purpose areas because they are often dominated by boys playing space-consuming games, as has been shown in other studies (Borve and Borve 2017; Karsten 2003; Shilling 1991). In her project, Karsten (2003) interviewed children playing in playgrounds and revealed that girls preferred good-quality playgrounds and challenging play objects like high climbing frames or big swings. A study of primary school children in Germany showed that attractively designed school playgrounds were positively related to girls’ regular overall physical activity (Möhrle et al. 2015). Additionally, Anthamatten et al. (2014) showed that girls were more likely to play in playground zones with equipment while boys were more active in those without equipment, such as playing field areas. Other studies have also observed gendered activities in playgrounds (Paechter and Clark 2007): Girls preferred playing with, at or inside play objects like huts, and boys preferred ball games such as soccer and tended to occupy larger spaces than girls did. However, in the present study we did not analyze types of play activity or where the children played in the playgrounds.

In both boys and girls, usage was higher in playgrounds providing more varied facilities, independent of playground size, and the quality and cleanliness of the play facilities. Thus, playgrounds with many different play facilities seem to draw more users, as also shown in previous studies (Colabianchi et al. 2011; Kaczynski et al. 2008). Also the number of children being at least moderately active was higher in playgrounds with more varied facilities in the adjusted models. In a systematic review, Broekhuizen et al. (2014) summarized seven studies and found a conclusive relationship between fixed equipment and permanent play facilities/improvements in school playgrounds (excluding pre-schools) and children’s physical activity levels. In another study investigating 5- to 12-year-old school children, the average activity counts measured by accelerometry increased by 3.8% during schooltime and 2.7% overall for each additional play facility (range 14–35 play facilities) (Nielsen et al. 2010). In summary, it can be said that the MVPA of children in playgrounds could be improved with the number of (varied) play facilities. However, playground density (children per square meter) may also have a negative effect on physical activity levels during recess (Ridgers et al. 2010) and on overall physical activity in boys and girls (Möhrle et al. 2015). Thus, increases in the number of play facilities may lead to an increased number of children using the playground, which may result in decreased physical activity if the play space per child is too small.

In our study, playground naturalness was inversely associated with playground usage in both genders and with MVPA in boys. This finding is surprising, as evidence suggests that children desire natural environments that provide opportunities for a wide range of different play and exploratory activities (Taylor et al. 1998; Tranter and Malone 2004). However, when investigating play choices of pre-school children in playgrounds of four Australian child care centers, Dyment and O’Connell (2013) found different play choices were made. While in two playgrounds the children partly preferred the more exciting natural areas, in another playground they preferred the paved areas. In particular, the boys were more likely to play in paved areas where they could engage better in functional play, bike riding and rule-bound games. Naturalness of playgrounds could offer additional opportunities for children and enhance their attractiveness. Nevertheless, some games and play activities like ball games, which are especially popular with boys, are bound to paved or non-natural surroundings.

Finally, fostering of physical activity levels in children is not the only rationale for public playgrounds as they are also designed to support social interaction, social development and development of fundamental motor skills, which have not been considered in the current study (Quigg et al. 2012). Spatial and material conditions in playgrounds to encourage these skills might be different from the features necessary to foster physical activity in playgrounds.

### Strengths and limitations

The strength of the current study is the investigation of playground usage and physical activity by children in neighborhood playgrounds in a non-school environment and out of schooltime. This study also takes into account gender differences and provides real-time data by observation of physical activity in the playgrounds. We were able to link playground features to physical activity, and we did not simply measure overall physical activity like in previous studies (Möhrle et al. 2015; Nielsen et al. 2012). By using an audit instrument as well as data from the city’s playground manual, we were able to capture a wide range of environmental data that included quantitative (e.g., play facility provision) and qualitative (e.g., cleanliness and esthetics) aspects of playground features. Nevertheless, this study is not without limitations. First, only ten playgrounds in one district of a middle-sized German town were assessed, on weekdays only and in the summertime of a single year. Thus, these results are limited in their generality. Second, misjudgements could have occurred because of difficulties in estimating the age and gender of the playground users by subjective assessment using human observers, and as we did not capture the personal data of the observed children, we were not able to analyze the relevance of socioeconomic status or migration backgrounds. Third, the observation activities themselves might have affected the children and their behavior. Finally, we did not consider social-environmental determinants of physical activity in this study although playground usage and physical activity levels of children in playgrounds are probably influenced by factors such as parental or peer modeling and support.

### Conclusion

The current study showed that usage and physical activity levels differed between playgrounds and that playground spatial features were related to children’s playground usage and activity levels. We suggest that playgrounds should offer a wide variety of play facilities and provide spaces (e.g., paved areas) for different play activities like ball games and objects to play with, such as huts, to respond to the needs of as many children of both genders as possible.
